# Successful management of Influenza A associated fulminant myocarditis: mobile circulatory support in intensive care unit: a case report

**DOI:** 10.1186/1757-1626-1-46

**Published:** 2008-07-18

**Authors:** Ambroise Montcriol, Sandrine Wiramus, Alberto Ribeiri, Nathalie Attard, Lyacine Nait-Saidi, François Kerbaul, Laurent Chiche

**Affiliations:** 1 Department of anesthesia and intensive care unit, La Timone Hospital, Saint Pierre street 264, 13885, Marseille, Cedex 5, France; 2 Department of anesthesia and intensive care unit, Sainte Marguerite Hospital, Bd sainte Marguerite 270, 13274, Marseille, France; 3 Department of cardiac surgery, La Timone Hospital, Saint Pierre street 264, 13885, Marseille, Cedex 5, France; 4 Department of cardiology, La Timone Hospital, Saint Pierre street 264, 13885, Marseille, Cedex 5, France

## Abstract

A 26-year-old woman was referred to an Emergency Department because of common flu-like syndrome with hemodynamic collapse. In Intensive Care Unit (ICU), she was diagnosed as a probable septic shock. But despite treatment her condition rapidly deteriorated during the subsequent hours. Diagnosis of cardiogenic shock was established. Mechanical circulatory support was inserted into the patient. She was transferred in a Cardio-Vascular Surgical ICU where at the 5^th ^day of mechanical circulatory support, echocardiography showed heart recovery which allowed weaning of mechanical circulatory support and progressive withdrawal of inotropic support. She was discharged at the 26^th ^day. During her hospitalization, presence of Influenza A RNA was shown in myocardial biopsy.

## Introduction

Fulminant myocarditis causing severe hemodynamic dysfunction and requiring high-dose vasopressors or mechanical circulatory support is rare; however, intensive care unit physicians should be aware that its treatment may require the use of mobile circulatory support devices. We report a case of acute fulminant myocarditis caused by influenza A infection, a rare etiology, successfully treated by percutaneous extracorporeal membrane oxygenation (ECMO) with cardio-pulmonary bypass.

## Case report

A previously healthy 26-year-old woman was referred to the Emergency Department with an influenza-like illness, which had started 2 days previously, and was associated with progressive disturbance of consciousness. Clinical examination was normal except for a blood pressure of 70/30 mmHg and a tachycardia of 120 beats/min. There was no response to a fluid challenge (1 L of colloid over 30 min). Central nervous system infection was ruled out by lumbar puncture and cerebral tomodensitometry. Her white blood cell count was 7,000 per μl and the procalcitonin concentration was not raised. EKG and chest radiography were normal. The first troponin measurement was 0.02 ng/mL.

The patient was transferred in a medical Intensive Care Unit (ICU) under the diagnosis of probable septic shock. Fluid resuscitation (4 L of cristalloids during the 6 first hours) was followed by a daily continuous infusion of 3 L of cristalloids and norepinephrine infusion. Antibiotics were given: cefotaxime (4 × 2 g/day, erythromycin 3 × 1 g/day and gentamycin 1 × 400 mg/day), plus intravenous hydrocortisone (200 mg per day).

Fluid resuscitation produced no improvement in blood pressure. Degradation of the oxygenation (PaO2/FiO2 < 120) requiring mechanical ventilation was observed. Her hemodynamic status rapidly deteriorated during the subsequent hours despite high doses of vasopressor (norepinephrine 5 mg/h). Diagnosis of cardiogenic shock was established by the association of severe hemodynamic compromise requiring high-dose of vasopressor, low right atrial oxygen saturation (45%) and transthoracic echocardiography showing a diffuse hypokinesia with an estimated left ventricular ejection fraction (LVEF) of 20%, elevated right and left filling pressures. Left ventricle diastolic dimensions were however normal (44 mm) as was septal thickness (8 mm). The troponin I concentration was 28 ng/mL. Monitoring by pulse contour analysis and thermodilution showed a cardiac index of 2.4 L/min/m^2^. Restriction of parenteral fluids and continuous veno-venous hemofiltration with fluid removal at an average rate of 300 ml/h substantially improved gas exchange. However, despite increased inotropic support (epinephrine 2 to 25 mg/h and dobutamine 12 μg/kg/min), the hemodynamic status further worsened and the multi-organ dysfunction syndrome became obvious (arterial lactate level at 10 mmol/L, alanine aminotransferase level of 293 IU/L, aspartate aminotransferase level of 587 IU/L, bilirubin level of 21 mg/dL and progressive oliguria) (Table 1).

**Table 1:** Evolution of hemodynamic data, inotropic support and biology at the admission and during hospitalization in medical ICU

		H	H+6	H+12	H+18	H+24	H+48
	
	Emergency department	ICU admission	ventilation	Before CVVHDF	Before ECMO		Before transfer in surgical ICU
**Hemodynamic data**							
MAP (mmHg)	60	66	80	78	55	78	86
Cardiac index (L × min^-1 ^× m^-2^)			4.4	3.8	2.4		
LVEF (%)			45	22	11		
Urinary output (mL/h)		450	400	33	40	80	100
**Inotropic support**							
Epinephrine (mg/h)				5	25	10	4
Norepinephrine (mg/h)		2.1	4.8	14.4	0		
Dobutamine (μg × kg^-1 ^× min^-1^)					8	13	17
**Biology**							
Troponin I (ng/mL)	0.75		10.10	18.6	29.77	28.36	23.18
BNP (pg/mL))				228		321	288
pH		7.36	7.16	7.27	7.19	7.13	7.30
Lactate level (mmol/L)	0.7	1.25	1.8	2.83	9.84	13.47	11.69
PaO2/FiO2		120	157	59	225	196	312
ASAT (IU/L)/ALAT (IU/L)	43/18	35/21		28/36	587/293		
Total bilirubin (mg/dL)		11		15	21		
INR	1.33			1.96	2.35		4.15
Factor V (%)				30%			< 20%

ICU: intensive care unit; CVVHDF: continuous veno-venous hemodiafiltration; ECMO: extracorporeal membrane oxygenation; MAP: mean arterial pressure; LVEF: left ventricle ejection fraction; BNP: brain natriuretic peptide; ASAT: aspartate aminotransferase; ALAT: alanine aminotransferase; INR International Normalized Ratio

A circulatory support device was connected by percutaneous femoral vein and artery cannulation. Pump flow of 3500 rpm generated an output of 4 L/min and a non pulsatile systemic pressure of 80 mmHg. Intravenous heparin was given, aiming at an activated cephalin time of 60–80 secs. This procedure allowed rapid weaning of the inotropic support, and led to urine production and decreased lactacidemia (Table 1).

The patient was transferred to a Cardiac ICU 24 hours later. Echocardiography showed lower left and right heart pressures, but the LVEF was persistently low at 20%. Multiple biopsies of the myocardium were performed. These latter, using routine staining, revealed interstitial inflammation and tissue destruction, but bacterial cultures remained negative. Real time polymerase chain reaction showed the presence of Influenza A RNA in the biopsy. Influenza A infection was confirmed by serologic tests and positive culture of the endotracheal aspirate. Liver function tests improved during circulatory support but there was anuric renal failure. On the 5^th ^day of mechanical circulatory support, echocardiography showed improved diastolic and systolic function (LVEF of 50%) which allowed the progressive withdrawal of inotropic support. Mechanical circulatory support was reduced on the 6^th ^day, and was withdrawn 24 hours later. The empiric antibiotherapy was stopped on the 7^th ^day; she was extubated on the 13^th^, and vasopressor support was withdrawn on the 15^th^. Renal function recovered after 20 days of continuous veno-venous hemofiltration. She was discharged on the 26^th ^day with an LVEF of 70%, good diastolic function and no ventricular dilatation.

## Discussion

Myocarditis is an inflammation of the myocardium caused by viral, bacterial or protozoal infections, drug toxicity or immunological reactions. Viral myocarditis remains the prototype for the study of this disease and its evolution. Nevertheless few cases of Influenza-associated myocarditis have been reported [1-6].

The diagnosis of myocarditis may be difficult to make in the early stages of the disease. In this case, it was initially misdiagnosed as septic shock, in which myocardial dysfunction and mild troponin increase are commonly seen. This case report underlines the role of echocardiography for diagnosis and management of myocarditis [7]. Left ventricular dysfunction is the most common abnormality reported during myocarditis but is unspecific. An increase in brightness, heterogeneity and contrast of the myocardium may suggest myocarditis. Furthermore, fulminant myocarditis could be distinguished from acute myocarditis by echocardiographic criteria. Patients with fulminant myocarditis typically have near-normal left ventricle diastolic dimensions with increased septal thickness at presentation, whereas those with acute myocarditis have increased diastolic dimensions but normal septal thickness [7]. However, in this case report, septal thickness was normal. Endomyocardial biopsy is still considered by many to be the gold standard for diagnosing myocarditis. Drawbacks are that the results are delayed, and even if diagnosis is confirmed or an etiologic agent identified, no specific treatment recommendations for myocarditis can be made. Indeed, influenza antiviral agents like amantadine, rimadantine or neuraminidase inhibitors have not been tested in influenza-associated myocarditis and immune modulatory therapies have not proved themselves in controlled trials [8].

The treatment thus remains supportive. Intensive care physicians suspecting fulminant myocarditis must be prepared to use therapeutic options such as circulatory support before severe organ failure develops. Usually heart transplantation is delayed for weeks. This strategy is strongly supported by the fact that fulminant myocarditis are commonly reversible within a few days and have a better long-term prognosis than acute myocarditis [8].

Circulatory support is used to maintain cardiac output and organ perfusion, and to minimize the need for inotropic support until myocardial recovery. Alternatively it can be a bridge to long-term assist devices or heart transplantation. We choose a non-pulsatile, servo-regulated flow system driven by a centrifugal pump with a membrane oxygenator. It is relatively inexpensive, and can be set up within 30 min by percutaneous cannulation of the femoral vessels at the bedside, and can take over the function of both heart and lungs. Non-pulsatile blood flow is adequate for short term circulatory support (2–3 weeks) [9]. Organ dysfunction can be prevented by setting a flow rate sufficient to normalize indicators of circulatory failure such as pH, lactate, and SVO_2 _[9]. Left ventricular preload must be optimized and third space fluid reduced by normalizing oncotic pressure, avoiding excessive volume loading and using hemofiltration. It is essential to maintain sinus rhythm and to preserve left ventricular contractility with inotropic support, to prevent secondary myocardial damage and thrombotic complication induced by blood stagnation, even if systemic perfusion is maintained. Protective mechanical ventilation is maintained to oxygenate any blood delivered to the pulmonary vascular bed by residual myocardial activity, and to avoid atelectasis [10].

Complications associated with these devices are frequent. Then, even if these devices can be set up in an emergency unit, their routine management remains the role of specialized units. Furthermore, the decision to switch to a pulsatile paracorporeal or implantable systems allowing extended periods of support, or to proceed with heart transplantation before complications of long term extracorporeal circulatory support occur, remains one for experienced clinicians.

## Conclusion

The diagnosis of fulminant myocarditis may be difficult in the intensive care unit and identification of the viral agent is uncommon. Echocardiography is invaluable when the hemodynamic status worsens despite normally adequate treatment. A human outbreak of avian Influenza A H5N1 could see many cases of influenza-associated myocarditis and thus intensive care unit physicians should be aware of the possibilities of mobile closed chest cardiopulmonary bypass.

## Consent

Written informed consent was obtained from the patient for publication of this case report. A copy of the written consent is available for review by the Editor-in-Chief of this journal.

## Competing interests

The authors declare that they have no competing interests.

## Authors' contributions

AM, SW and NA wrote the manuscript, LNS performed echocardiography, AR performed surgical valve replacement, FK and LC corrected the manuscript. All authors read and approved the final manuscript.
